# Evaluating mitophagy in embryonic stem cells by using fluorescence-based imaging

**DOI:** 10.3389/fcell.2022.910464

**Published:** 2022-09-15

**Authors:** Kun Liu, Xing Li, Zheng Li, Jiani Cao, Xiaoyan Li, Youqing Xu, Lei Liu, Tongbiao Zhao

**Affiliations:** ^1^ State Key Laboratory of Stem Cell and Reproductive Biology, Institute for Stem Cell and Regeneration, Institute of Zoology, Chinese Academy of Sciences, Beijing, China; ^2^ University of Chinese Academy of Sciences, Beijing, China; ^3^ Beijing Institute for Stem Cell and Regenerative Medicine, Beijing, China; ^4^ Department of Gastroenterology, Beijing Tiantan Hospital, Capital Medical University, Beijing, China

**Keywords:** ATG3, LC3, mitophagy, mt-keima, PINK1

## Abstract

Embryonic stem cells (ESCs), which are characterized by the capacity for self-renewal and pluripotency, hold great promise for regenerative medicine. Increasing evidence points to the essential role of mitophagy in pluripotency regulation. Our recent work showed that PINK1/OPTN take part in guarding ESC mitochondrial homeostasis and pluripotency. Evaluating mitophagy in ESCs is important for exploring the relationships between mitochondrial homeostasis and pluripotency. ESCs are smaller in size than adult somatic cells and the mitophagosomes in ESCs are difficult to observe. Many methods have been employed—for example, detecting colocalization of LC3-II and mitochondria—to evaluate mitophagy in ESCs. However, it is important to define an objective way to detect mitophagy in ESCs. Here, we evaluated two commonly used fluorescence-based imaging methods to detect mitophagy in ESCs. By using autophagy- or mitophagy-defective ESC lines, we showed that the mito-Keima (mt-Keima) system is a suitable and effective way for detecting and quantifying mitophagy in ESCs. Our study provides evidence that mt-Keima is an effective tool to study mitophagy function in ESCs.

## Introduction

Mitochondria are double-membrane organelles which play vital roles in multiple biological processes, such as generating ATP, maintaining calcium homeostasis, regulating lipid metabolism and activating apoptosis (Levine and Klionsky, 2004; Zhu et al., 2011). Mitochondria undergo dynamic changes in response to the cell cycle and the physiological environment. Fission, fusion and mitophagy are three important ways of regulating mitochondrial homeostasis (Yoo and Jung, 2018). Reactive oxygen species (ROS) are primarily produced by mitochondria. ROS not only serve as signal molecules, they also trigger cellular oxidative damage and apoptosis. If mitochondria become dysfunctional, they will be degraded in a timely manner (Wei et al., 2015). Mitophagy is the specific process for elimination of damaged or excessive mitochondria by lysosome-dependent degradation.

Pluripotent stem cells (PSCs) include embryonic stem cells (ESCs) and induced pluripotent stem cells (iPSCs) (Liu et al., 2014). Due to their indefinite self-renewal and the ability to differentiate into all cell types of the 3 germ layers, PSCs are valuable cell resources for research and clinical applications (Martello and Smith, 2014). The mitochondria in PSCs are small, spherical and less developed compared to the ones in differentiated cells, which are elongated and well developed with dense cristae. Although mitochondria have simplified structures in PSCs, they can still balance self-renewal and pluripotency (Facucho-Oliveira et al., 2007; Yoo and Jung, 2018; Todd et al., 2010; Folmes et al., 2016). Compared with fibroblasts, PSCs have a high proliferation rate and a short cell cycle. This means that mitochondria should be recycled and generated quickly in PSCs. Dysfunctional and excessive mitochondria will arise during cell division. Removal of dysfunctional mitochondria is necessary for avoiding oxidative damage in ESCs. However, there is no efficient way for detecting mitophagy in ESCs. Our recent work revealed that high autophagy flux was required for efficient degradation of damaged organelles and aggregated proteins in ESCs (Liu et al., 2017). Furthermore, we explored whether autophagy was responsible for mitochondrial degradation, and how mitophagy is regulated in ESCs. By using an Atg3 knockout model and a Pink1/Bnip3 knockout model, we identified that mitochondrial autophagy is essential for maintaining the identity of ESCs (Liu et al., 2016), and both Pink1 and Bnip3-mediated mitophagy maintains the mitochondrial homeostasis in ESCs (Liu et al., 2022; Wang et al., 2021). However, the mechanisms by which mitophagy regulates pluripotency are still largely unknown. One of the limiting factors is the lack of a proper method for monitoring mitophagy in ESCs.

**FIGURE 1:**
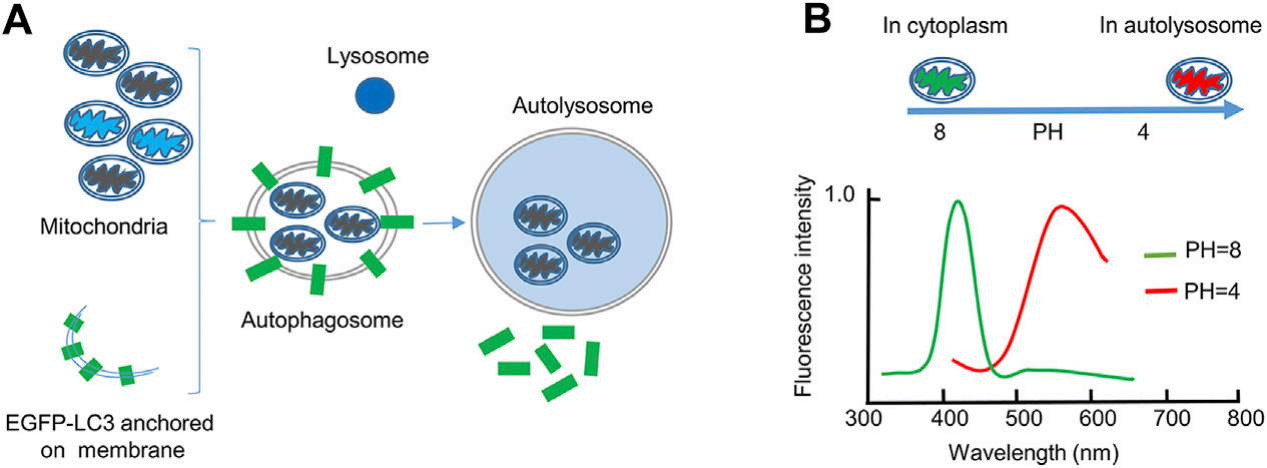
The EGFP-LC3 and mt-Keima systems for monitoring mitophagy. **(A)**, The EGFP-LC3 system. In this system, the autophagosomal marker protein LC3 is fused with EGFP. When mitochondria are dysfunctional (black), they will be engulfed by a double-membrane autophagosome for degradation. EGFP-LC3 is anchored on both the inner and outer membranes of autophagosomes. When mitochondria are stained with Mito-Tracker Red, colocalization of the two fluorescent signals indicates mitophagy. After the autophagosome fuses with the lysosome, the EGFP signal will disappear, because the EGFP-LC3 returns to the cytoplasm or is degraded by lysosomal proteases. **(B)**, The mt-Keima system. In this system, the fluorescent protein Keima, derived from coral, is fused with a mitochondrial matrix protein. The excitation wavelength of mt-Keima is sensitive to pH. At neutral pH, the excitation wavelength of mt-Keima is 440 nm; therefore, it can be detected by 458 excitation laser. At acidic pH, the excitation wavelength of mt-Keima is 586 nm; therefore, it can be detected by 561 excitation laser. The lysosomal lumen is an acidic environment, and therefore mitochondria engulfed in autolysosomes can be monitored by 561 excitation laser.

Electron microscopy is the classical method for detecting mitophagy in cells and tissues. However, this method needs an experienced investigator to observe autophagic membranes engulfing or surrounding mitochondria. Sample observation requires sophisticated microscopes and experienced technicians. In addition, it is tedious to use this technique to quantify the mitophagosome changes. These disadvantages limit the wide usage of electron microscopy (Sun et al., 2017). In contrast, using fluorescence-based imaging is convenient and efficient. Therefore, fluorescent mitochondrial and autophagic markers are used together for monitoring mitophagy (Figure 1A). Old, damaged or dysfunctional mitochondria are selectively recognized and engulfed by autophagosomes. The autophagosomes then fuse with lysosomes for degradation. Usually, the occurrence of mitophagy is indicated by the colocalization between mitochondrial markers and the autophagosome-specific marker LC3 (Dolman et al., 2013). Recently a protein probe called mito-Keima (mt-Keima) has been used for measuring mitophagy by fluorescence microscopy. It is convenient, sensitive and easier to quantify (Figure 1B). Keima is a pH-sensitive and dual-excitation ratiometric fluorescent protein. Most importantly, it is resistant to lysosomal proteases (Katayama et al., 2011). The emission spectrum peak of Keima is 620 nm. Keima has a bimodal excitation spectrum peaking at 440 and 586 nm, respectively, in neutral and acidic pHs (Violot et al., 2009). Mt-Keima is a powerful tool to monitor mitophagy both *in vitro* and *in vivo*. Previous studies have shown that mt-Keima functions in somatic cells and different tissues (Sun et al., 2015; Shirakabe et al., 2016; Sun et al., 2017). However, ESCs are small in size and undergo rapid division and clone-like growth; these qualities make them very different from most cell types. Therefore, monitoring the mitophagy in ESCs is difficult. It is unknown whether the LC3/mitochondrion colocalization system or the mt-Keima system can be used in ESCs. Which of them is most convenient and efficient for detecting and quantifying mitophagy in ESCs? Here, we evaluated the two fluorescence-based systems and compared their ability to detect mitophagy in ESCs. Finally, we identified that mt-Keima is most advantageous for quantifying mitophagy in ESCs.

## Results

### Mitophagy activation in the embryonic stem cell system

**FIGURE 2:**
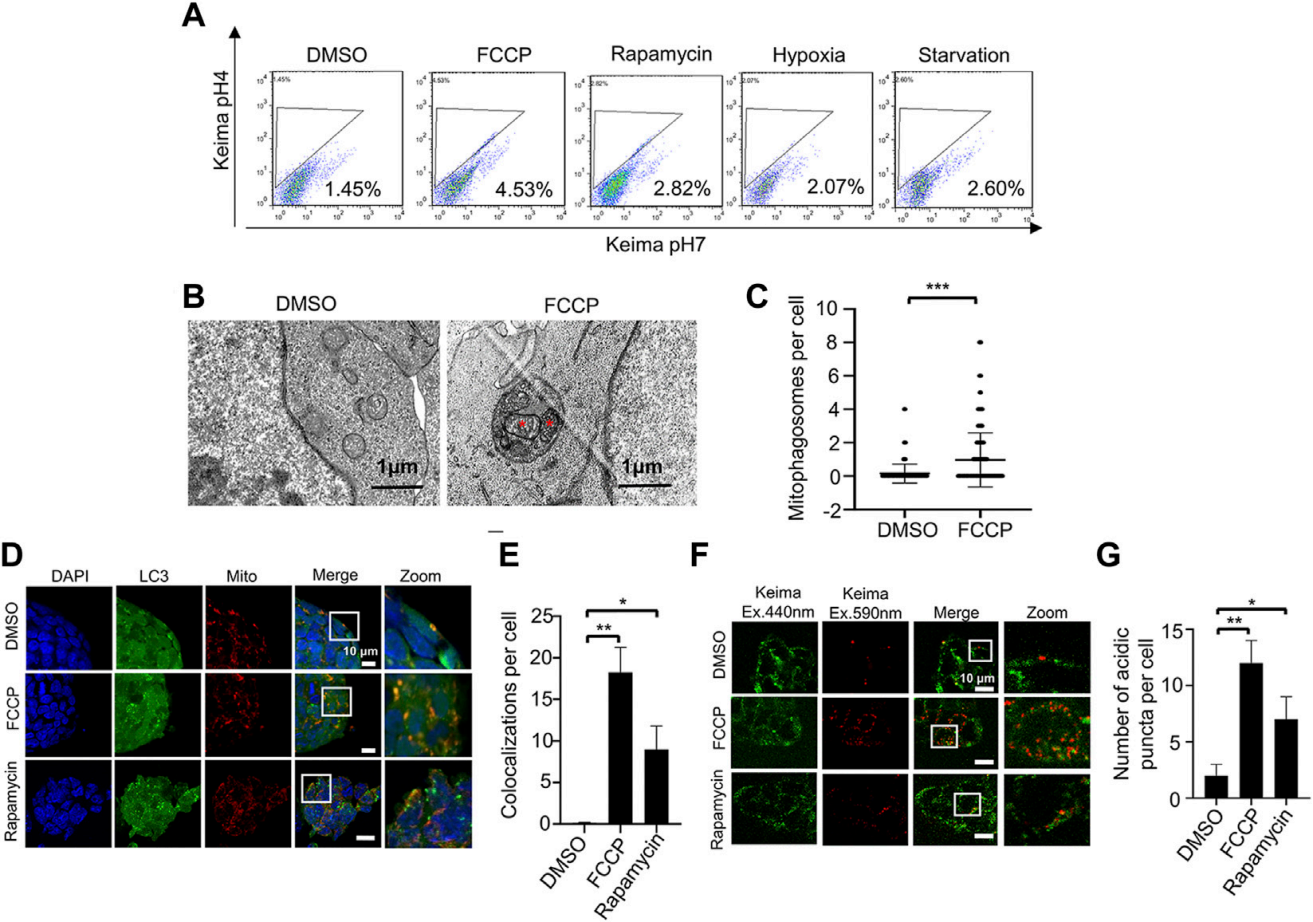
Two fluorescence-based imaging systems for measuring mitophagy in ESCs under mitochondrial stress. **(A)**, Mitophagy activation in ESCs is evaluated by mt-Keima system using a FACS. ESCs stably expressing mt-Keima were treated with FCCP, rapamycin, hypoxia or starvation, and then analyzed by a FACS. Data shown is one of three independent experiments. **(B)**, FCCP triggers mitophagy in ESCs. ESCs were treated without or with 10 nM FCCP, then samples were harvested for examination by transmission electronic microscopy. The red asterisks indicate two mitochondria enclosed within an autophagosome. **(C)**, Quantifications of mitophagosomes in **(B)**. Mitophagosomes were counted in 200 cells. Data are shown as mean ± SD, *n* = 200; ***p < 0.001, Student’s t-test. **(D)**, The EGFP-LC3 system for monitoring mitophagy. By using Mito-RFP, EGFP-LC3 ESCs, we can observe co-localizations of LC3 and mitochondria. After FCCP or rapamycin treatment, co-localizations of LC3 and mitochondria are increased. Green: LC3; red: mitochondria; blue: DAPI. **(E)**, Quantification of mitophagy in ESCs by the EGFP-LC3 system. Co-localizations of LC3 and mitochondria were counted in 200 cells from 3 independent experiments. Data are shown as mean ± SD, *n* = 3; *p < 0.05, **p < 0.01, Student’s t-test. **(F)**, The mt-Keima system for monitoring mitophagy. By transfecting mt-Keima into ESCs, we can observe the mitochondrial network (green signal) and mitochondria within acidic autolysosomes (red puncta). After adding FCCP or rapamycin, the number of red puncta increases. **(G)**, Quantification of mitophagy in ESCs by the mt-Keima system. Red puncta were counted in 200 cells from 3 independent experiments. Data are shown as mean ± SD, *n* = 3; *p < 0.05, **p < 0.01, Student’s t-test.

To investigate the mitophagy activation, we treated ESCs with different stimulators, including FCCP (carbonyl cyanide-4 (trifluoromethoxy) phenylhydrazone), rapamycin, hypoxia and starvation. We detected the mitophagy related markers by western blot. Data showed that the expression of mitophagy marker Pink1, OPTN and LC3 were increased, with no significant changes in mitochondrial protein TIM23 (Supplementary Figure S1). It might due to low mitochondrial content in ESCs, and western blot was not sensitive enough to detect the slightly change of mitochondrial protein. Previous study showed that FACS of mt-Keima stained cells was a sensitive and quantitative assay for measuring mitophagy (Wang, 2020). Therefore, we transfected mt-Keima plasmid into ESCs and measured mitophagy activation under different treatments by FACS. Data showed that all treatments could activate mitophagy in ESCs, with FCCP and rapamycin treatments in a more efficient way (Figure 2A). We also performed ratiometric analysis of mt-Keima, and the result was consistent with FACS data (Supplementary Figure S2).

### FCCP triggers mitophagy in embryonic stem cells

In order to confirm mitophagy in ESCs, we firstly treated ESCs with FCCP to trigger mitophagy. By using transmission electron microscopy, we observed mitochondrion-containing autophagosomes (mitophagosomes) in the drug-treated cells. In comparison, we detected no mitophagosomes in control cells (Figure 2B). Quantification of mitophagosomes in ESCs confirmed that drug treatment could significantly enhance mitophagy (Figure 2C). These data indicate that we have established specific cellular conditions for induction of mitophagy in ESCs.

### Detecting mitophagy by the EGFP-LC3 system

The EGFP-LC3 system is the common way to evaluate mitophagy, based on measuring the colocalization of mitochondria (labeled with Mito-Tracker Red) and autophagosomes (labeled with EGFP-LC3). We firstly tested whether this system can work in ESCs. Under normal conditions (no FCCP and rapamycin), we found only rare colocalization of mitochondria and autophagosomes. After treatment with FCCP or rapamycin, the colocalization of mitochondria and autophagosomes increased significantly (Figure 2 D,E). It is easy to distinguish the colocalized signals when the ESCs are cultured in normal conditions. However, when the ESCs are stressed (in the presence of FCCP or rapamycin), the overlapping fluorescent signals are less distinct, so it is more difficult to quantify the colocalization of mitochondria and autophagosomes. In addition, LC3 can aggregate in an autophagy-independent way, which would increase the false positive rate (Kuma et al., 2007). Therefore, we wanted to test other fluorescence-based imaging systems to monitor mitophagy in ESCs.

### Detecting mitophagy by the mt-Keima system

Mt-Keima is a newly developed probe, which has two distinct excitation spectrums and a single emission spectrum. Most importantly, mt-Keima has a shorter wavelength (green) excitation in physiological pH, while it has a longer wavelength (red) excitation in acid pH. This means that mitochondria can be monitored in the cytosol (physiological pH) and in autophagosomes/lysosomes (acid pH) by the fluorescence shift. We transfected the mt-Keima plasmid into ESCs, and tested whether it could work in these cells. We used FCCP or rapamycin to trigger mitophagy as described before, and we observed numerous red puncta. This means that mitochondria were delivered into an acidic environment. By counting the red puncta, we found that the number of mitochondria in lysosomes was significantly increased (Figure 2 F,G). Compared with the EGFP-LC3 system, it is easy to measure mitophagy with the mt-Keima system, and the cell size does not have much effect.

### Detecting mitophagy in Atg3 knockout embryonic stem cells by the two fluorescence-based imaging methods

**FIGURE 3:**
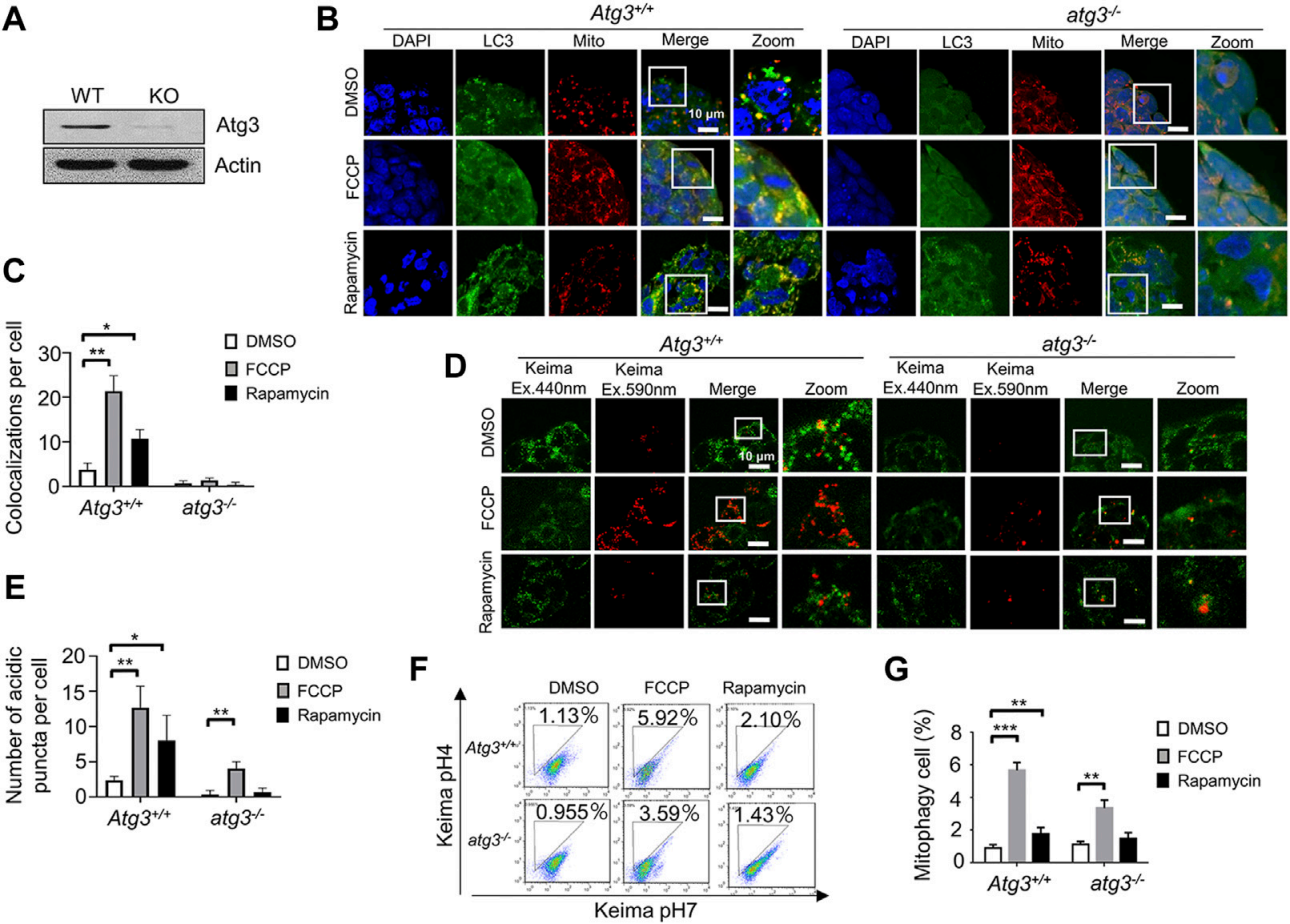
Detecting mitophagy in Atg3 knockout ESCs by the two fluorescence-based imaging systems. **(A)**, Western blot of Atg3 wild-type and knockout ESCs. Actin serves as a loading control. **(B)**, Monitoring mitophagy in Atg3 knockout ESCs by the EGFP-LC3 system. After treatment with FCCP or rapamycin, the co-localizations of LC3 and mitochondria increased greatly in wild-type ESCs, while there were very few co-localizations in the knockout ESCs. Green: LC3; red: mitochondria; blue: DAPI. **(C)**, Quantification of mitophagy in **(B)**. Co-localizations of LC3 and mitochondria were counted in 200 cells from 3 independent experiments. Data are shown as mean ± SD, *n* = 3; *p < 0.05, **p < 0.01, Student’s t-test. **(D)**, Monitoring mitophagy in Atg3 knockout ESCs by the mt-Keima system. The number of mitophagy puncta is increased in both Atg3 wild-type and knockout ESCs upon FCCP treatment. **(E)**, Quantification of mitophagy in **(D)**. The number of mitophagy puncta increased significantly in Atg3 wild-type and knockout ESCs upon FCCP treatment. Red puncta were counted in 200 cells from 3 independent experiments. Data are shown as mean ± SD, *n* = 3; *p < 0.05, **p < 0.01, Student’s t-test. **(F)**, Evaluation of mitophagy in Atg3 wild-type and knockout ESCs by a FACS. The number of mitophagy cell is increased in Atg3 wild-type ESCs upon FCCP or rapamycin treatment. **(G)**, Quantifications of mitophagy cell in **(F)**. Data are shown as mean ± SD, n = 3; **p < 0.01, ***p < 0.001, Student’s t-test.

Autophagy-related genes are required for autophagosome formation. Atg3, working as a membrane-sensor, plays a critical role in autophagosome formation. We further tested whether blocking autophagy would impair mitophagy in ESCs. We isolated Atg3 wild-type and knockout ESCs from mice, and validated them by western blot (Figure 3A). By using the EGFP-LC3 system, we found that Atg3 deficiency significantly decreased the colocalization of mitochondria and autophagosomes. In Atg3 knockout ESCs, EGFP-LC3 puncta were rare and hard to detect (Figures 3B,C). At the same time, we used the mt-Keima system to detect the mitophagy in Atg3 wild-type and knockout ESCs. The red puncta were clear enough to count in both Atg3 wild-type (WT) and knockout ESCs. By counting the red puncta, we found that FCCP induced a significant increase of mitophagy in both Atg3 wild-type and knockout ESCs. In contrast, rapamycin-induced mitophagy can be detected in WT but not in knockout ESCs (Figure 3 D,E). We confirmed our fluorescence-based imaging data by a FACS assay (Figure 3 F,G).

### Detecting mitophagy in Pink1 knockout embryonic stem cells by the two fluorescence-based imaging methods

**FIGURE 4:**
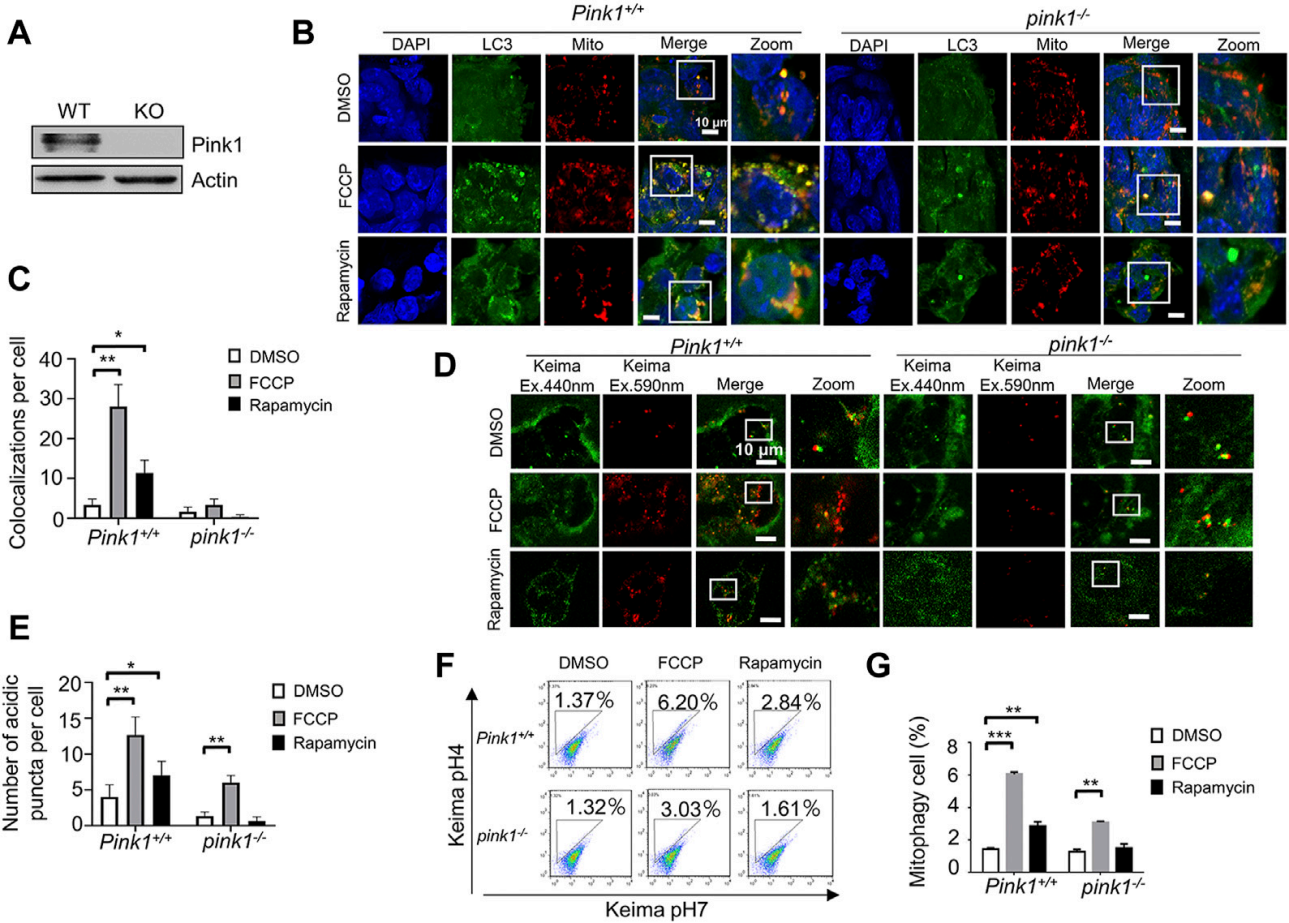
Detecting mitophagy in Pink1 knockout ESCs by the two fluorescence-based imaging systems. **(A)**, Western blot of Pink1 wild-type and knockout ESCs. **(B)**, Monitoring mitophagy in Pink1 wild-type and knockout ESCs by the EGFP-LC3 system. After FCCP or rapamycin treatment, the co-localizations of LC3 and mitochondria clearly increased in wild-type ESCs, but not in knockout ESCs. Green: LC3; red: mitochondria; blue: DAPI. **(C)**, Quantification of mitophagy in **(B)**. Co-localizations of LC3 and mitochondria were counted in 200 cells from 3 independent experiments. Data are shown as mean ± SD, *n* = 3; *p < 0.05, **p < 0.01, Student’s t-test. **(D)**, Monitoring mitophagy by the mt-Keima system. More mitophagy puncta were observed in both Pink1 wild-type and knockout ESCs following FCCP treatment. **(E)**, Quantification of mitophagy in **(D)**. Red puncta were counted in 200 cells from 3 independent experiments. Data are shown as mean ± SD, *n* = 3; *p < 0.05, **p < 0.01, Student’s t-test. **(F)**, Evaluation of mitophagy in Pink1 wild-type and knockout ESCs by a FACS. **(G)**, Quantifications of mitophagy cell in **(F)**. Data are shown as mean ± SD, *n* = 3; **p < 0.01, ***p < 0.001, Student’s t-test.

Our recent work revealed that Pink1 is essential for mitophagy in ESCs (Wang et al., 2021). Here, we used the Pink1 knockout mouse model to evaluate which fluorescence-based imaging system is more suitable for monitoring mitophagy in ESCs. Firstly, we isolated Pink1 knockout ESCs from the mice and validated them by western blot (Figure 4A). Secondly, we stained Pink1 wild-type and knockout ESCs by Mito-tracker and anti-LC3 antibody. By using confocal microscopy, we found that the levels of mitophagy were significantly increased in FCCP or rapamycin-treated wild-type ESCs compared to control wild-type ESCs. However, there was no significant change in FCCP or rapamycin-treated Pink1 knockout ESCs compared to untreated Pink1 knockout ESCs (Figure 4 B,C). In contrast, using the mt-Keima system, we observed a significantly increased number of acidic mitochondrial puncta in Pink1 knockout ESCs after FCCP treatment (Figure 4 D,E). The FACS data confirmed the fluorescence-based imaging results (Figure 4 F,G).

### The mt-keima system is more suitable for detecting mitophagy in embryonic stem cells

To compare the utility of the two methods, we used them to measure mitophagy in Atg3 knockout and Pink1 knockout ESCs. With the EGFP-LC3 system, no significant increase in mitophagy was observed in the presence of FCCP or rapamycin in Atg3 or Pink1 knockout ESCs (Figure 3C, 4C). With the mt-Keima system, the significant increase of mitophagy puncta was detected after FCCP treatment, even though the level of mitophagy was much lower in Atg3 or Pink1 knockout ESCs compared to the wild-type ESCs (Figure 3E and Figure 4E). The mt-Keima system reveals that mitophagy can still occur in Atg3 or Pink1 knockout ESCs at a low level. Thus, mitochondrial degradation may occur in an Atg3-independent or Pink1-independent pathway. From these data, we think that the mt-Keima system is more suitable and solid than the EGFP-LC3 system.

### Non-canonical autophagy contributes to mitophagy in Atg3 or Pink1 knockout embryonic stem cells

Since mitophagy occurrence could be detected in Atg3 or Pink1 knockout ESCs by using mt-Keima system, indicating the existence ATG-independent mitophagy in ESCs, we used non-canonical autophagy inhibitor to treat the mitophagy induced Atg3 or Pink1 knockout ESCs. We found inhibition of non-canonical autophagy did inhibit mitophagy in PINK1 or ATG3 knock out ESCs (Supplementary Figure S3) (Ma et al., 2015). Our data suggest that non-canonical autophagy contributes to mitophagy in Atg3 and Pink1 knockout ESCs.

## Discussion

ESCs are pluripotent and have a high proliferation rate with a short G1 phase. They are regarded as potential biological material for regenerative medicine (White and Dalton, 2005; Ruiz et al., 2011). Mitochondria are important cellular organelles for energy production and signaling pathways. Although mitochondria are immature in ESCs, they are necessary for maintenance of stem cell identity (Folmes et al., 2016). When mtDNA-specific polymerases are knocked down, or if mitochondrial fission is promoted, the ESCs will lose pluripotency (Facucho-Oliveira et al., 2007; Todd et al., 2010). Recently, an interesting study showed that mitochondria can affect the fate of stem cells. This research revealed that the mitochondria divided asymmetrically in epithelial stem-like cells, producing two daughter cells with distinct fates (Katajisto et al., 2015). The daughter cells with fewer old mitochondria would maintain the stem cell trait, while the daughter cells receiving more aged mitochondria would lose their stem cell traits easily under stress. This means that the quality of mitochondria is critical for maintaining stem cell traits. Furthermore, our previous work revealed that both the quantity and quality of mitochondria are essential for stem cell identity. Autophagy is essential for degradation of proteins and organelles (Youle and Narendra, 2011). When autophagy is blocked, the dysfunctional mitochondria will accumulate in ESCs. Dysfunctional mitochondria increased the ROS in ESCs, and decreased the pluripotency of ESCs (Liu et al., 2016). These studies demonstrate that mitochondrial homeostasis is essential for identity maintenance in ESCs.

Mitophagy is responsible for regulating mitochondrial quantity and quality in cells. Somatic cells, such as neuronal cells and fibroblasts, have been widely used to monitor mitophagy (Chambers et al., 2013; Zhang and Ney, 2010; Zhu et al., 2011). However, the mitochondria in ESCs are small and less developed. Furthermore, ESCs are small, so it is not easy to monitor the dynamic changes of organelles and proteins within ESCs. These factors make it difficult to monitor and quantify the dynamic changes and regulation of mitochondria in ESCs. Here, we used two fluorescence-based imaging methods for detecting mitochondrial degradation, and we evaluated them in ESCs. We found that the mt-Keima system is more reliable than the EGFP-LC3 system. In Atg3 knockout ESCs or Pink1 knockout ESCs, the EGFP-LC3 system only detects a few puncta. The colocalized EGFP-LC3/mitochondrial puncta are hard to find. However, we can clearly find and quantify the red mitochondrial puncta in the mt-Keima system. More importantly, the mt-Keima system truly reflects the removal of mitochondria in ESCs. Comparing the two methods, we suggest that mt-Keima is more convenient and reliable for monitoring mitophagy in ESCs.

In previous studies, the most widely used method is electron microscopy, which can directly see the mitochondria engulfed by double membranes. However, electron microscopy has many limitations, such as the time-consuming sample preparation procedure, the possibility of poor sample orientation and the limited observation zone. It is difficult to measure mitophagy using this method, because capturing membrane-engulfed mitochondria needs experience and skill (Klionsky et al., 2016). Therefore, fluorescence-based imaging is widely used in most cell lines. However, ESCs have unique features, such as their small size and clone-like growth, which make it difficult to monitor mitophagy. By using fluorescence-based imaging, we found that the EGFP-LC3 system can work in ESCs. Due to the small cell size, it is difficult to distinguish the single EGFP-LC3 puncta from the colocalized puncta in ESCs. Using the mt-Keima system, we can avoid the high false positive rate, and truly evaluate the level of mitophagy in autophagy-defective ESCs. The only limitation of mito-Keima is that it must be used in living cells. Here, we evaluated the fluorescence-based imaging methods and identified the best one for measuring mitophagy.

Although ESCs have been investigated for decades, mitochondrial regulation in ESCs is still not clear. Developing a proper method is essential for exploring mitochondrial dynamic changes and homeostasis in ESCs. Our work offers a suitable technique for measuring mitophagy in ESCs. Remarkably, the mt-Keima system can monitor mitophagy in Atg3-or Pink1-deficient ESCs, and it will be helpful for comprehensively researching mitophagy and pluripotency regulation in ESCs.

## Materials and methods

### Animals, antibodies and reagents

B6D2-Tg (CAG/Su9-DsRed2, Acr3-EGFP) RBGS002Osb (RBRC03743) (Hasuwa et al., 2010), MAP1LC3B-GFP (RBRC00806) (Mizushima et al., 2004), and Atg3 heterozygous (RBRC02761) (Sou et al., 2008) mice were bought from Riken BioResource Center. Mito-Tracker Red (40743ES50) was bought from Yeasen. The anti-MAP1LC3B antibody (Medical and Biological Laboratories Co., PM036) was used at 1:200; Alexa Fluor^®^488 donkey anti-rabbit IgG (H + L) (Invitrogen Thermo Fisher Scientific, A21206) was used at 1:500; anti-Actin (1:5,000) was obtained from Sigma Aldrich (A5441); anti-ATG3 (1:500) was purchased from Cell Signaling Technology (3415S); anti-PINK1 polyclonal antibody (NOVUS, BC100–494) was used at 1:1,000; Anti-OPTN polyclonal antibody (1:1,000) was bought from Proteintech Group (10837-1-AP); FCCP (C2920) and EBSS (E2888) were obtained from Sigma Aldrich. Rapamycin (HY-10219) and Brefeldin A (HY-16592) were bought from Med Chem Express. Mito-Keima vector was shared by Prof. Chen Quan (Wu et al., 2020). Tim23 antibody (BD Biosciences, 611223) was shared by Prof. Liu Lei.

### Isolation and establishment of embryonic stem cells

B6D2-Tg RBGS002Osb mice were crossed with MAP1LC3B-GFP mice to obtain Mito-RFP, EGFP-LC3 ESCs. Atg3 heterozygous mice were used to isolate Atg3 wild-type and knockout ESCs. The ESCs were isolated at embryonic day 3.5 and cultured on feeders for 5 days in 2i medium. The ESC clones were picked and cultured for 3 to 5 passages. After that, ESCs were cultured in ESC medium as previously described (Liu et al., 2016). Pink1 knockout ESCs were established as previously described (Wang et al., 2021).

### Drug treatment of embryonic stem cells

For FCCP treatment, ESCs were cultured in ESC medium containing 10 nM FCCP for 24 h. For rapamycin treatment, ESCs were cultured in ESC medium containing 5 nM rapamycin for 24 h. For hypoxia, ESCs were cultured under hypoxia condition for 24 h. For starvation, ESCs were cultured in EBSS medium for 4–6 h. All above ESCs were harvest for western blot or FACS analysis.

### Electron microscopy

Samples were fixed in 2.5% glutaraldehyde for 2 h at 4°C, then immersed in 2% osmium tetroxide. After postfixation, the samples were dehydrated sequentially in 50, 70, 90, 95, and 100% ethanol. Samples were collected on copper grids, then counterstained with uranyl acetate and lead citrate. Images were taken by electron microscopy (JEM-1400) (Liu et al., 2017).

### Immunofluorescence microscopy

ESCs were cultured on gelatin-coated glass slides, and mitochondria were stained with Mito-Tracker Red (50 nM) for 30 min at room temperature. The cells were then fixed by 4% paraformaldehyde for 20 min, washed 3 times in Dulbecco’s PBS (DPBS), and treated with 0.2% Triton-X100 for 30 min. After that, the samples were blocked with 2% bovine serum albumin for 1 h, incubated with primary antibodies for 14 h at 4°C, washed with DPBS, and stained with the secondary antibody for 2 h at room temperature. Cell nuclei were stained with DAPI. For mito-Keima system imaging, we packaged lentiviruses and infected ESCs, then directly observed the cells under a confocal microscope (Zeiss LSM880 Fast Ariyscan) without fixing.

### Western blotting

Cells were lysed with lysis buffer (50 mM Tris, pH 7.4, 2 mM EDTA, 1% NP-40, protease inhibitors). Equivalent protein quantities were subjected to SDS-PAGE, and transferred to nitrocellulose membranes. After blocking with 5% milk, membranes were incubated with primary antibodies, followed by the appropriate HRP-conjugated secondary antibodies. Finally, immunoreactive bands were detected by Luminata Forte Western HRP Substrate.

### FACS-based mKeima assay

We transfected mt-Keima into ESCs by Lenti-virus (Wu et al., 2020), and then sorted stably expressing mt-Keima ESCs by a FACS. After that, we treated ESCs containing mt-Keima with FCCP, rapamycin, hypoxia or starvation for 24 h. All above ESCs were harvest for FACS analysis. We harvested 10000 cells in gated and conducted the analysis with reference to the method of Wang et al. (Wang, 2020). In this experiment, every sample had three duplicates. We had repeated each experiment at least three times.

### Statistical analysis

Data were compared by Student’s t-test using SPSS. *p* < 0.05 was considered as statistically significant.

## Data Availability

The original contributions presented in the study are included in the article/Supplementary Material, further inquiries can be directed to the corresponding author.
