# Supplementary figures and images for: Evaluating mitophagy in embryonic stem cells by using fluorescence-based imaging
Source: Front Cell Dev Biol. 2022 Sep 15;10:910464. doi: 10.3389/fcell.2022.910464 (PMC9520453; doi:10.3389/fcell.2022.910464)

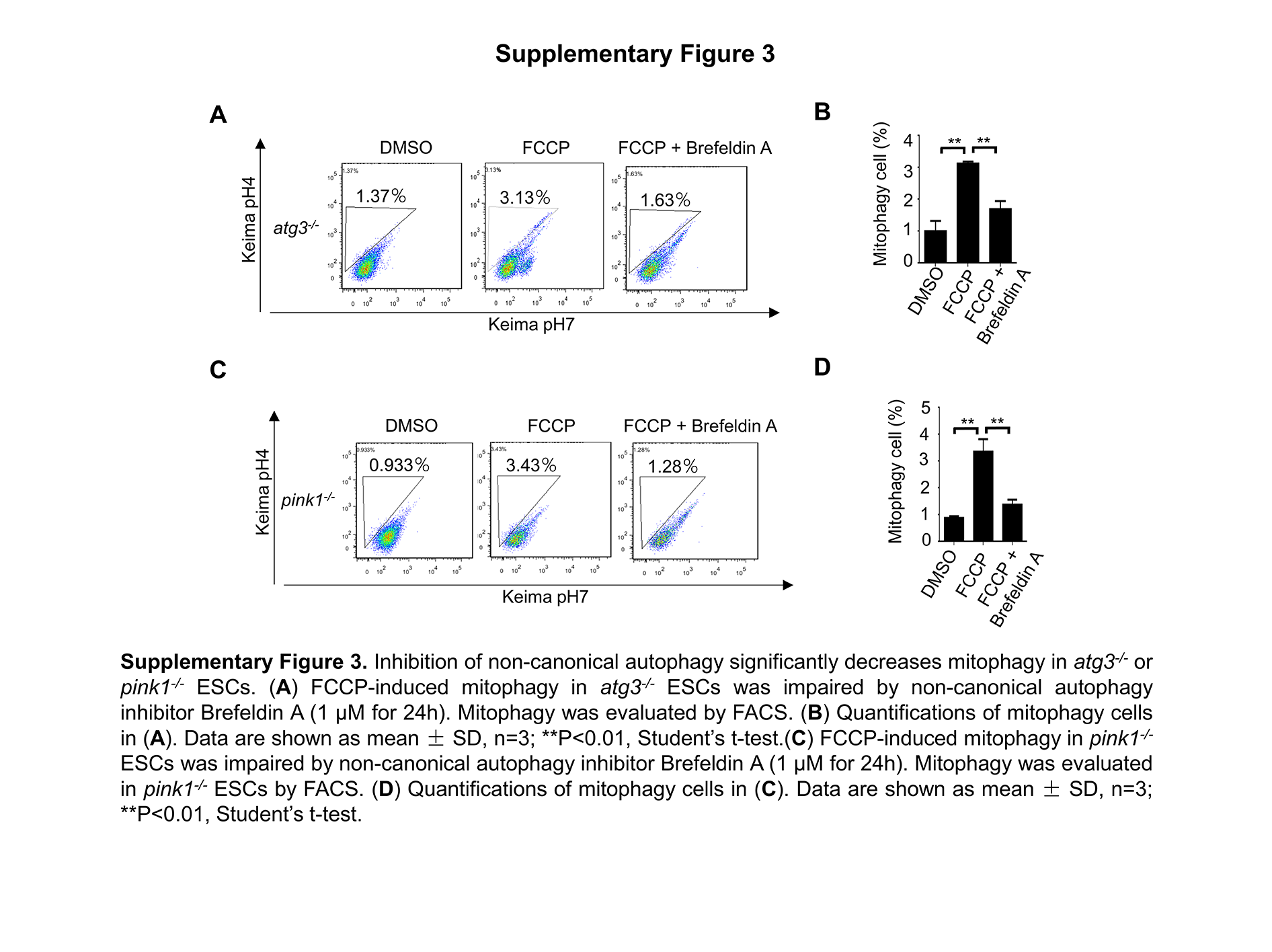

Supplement: Supplementary file 1 [file Image3.TIF]

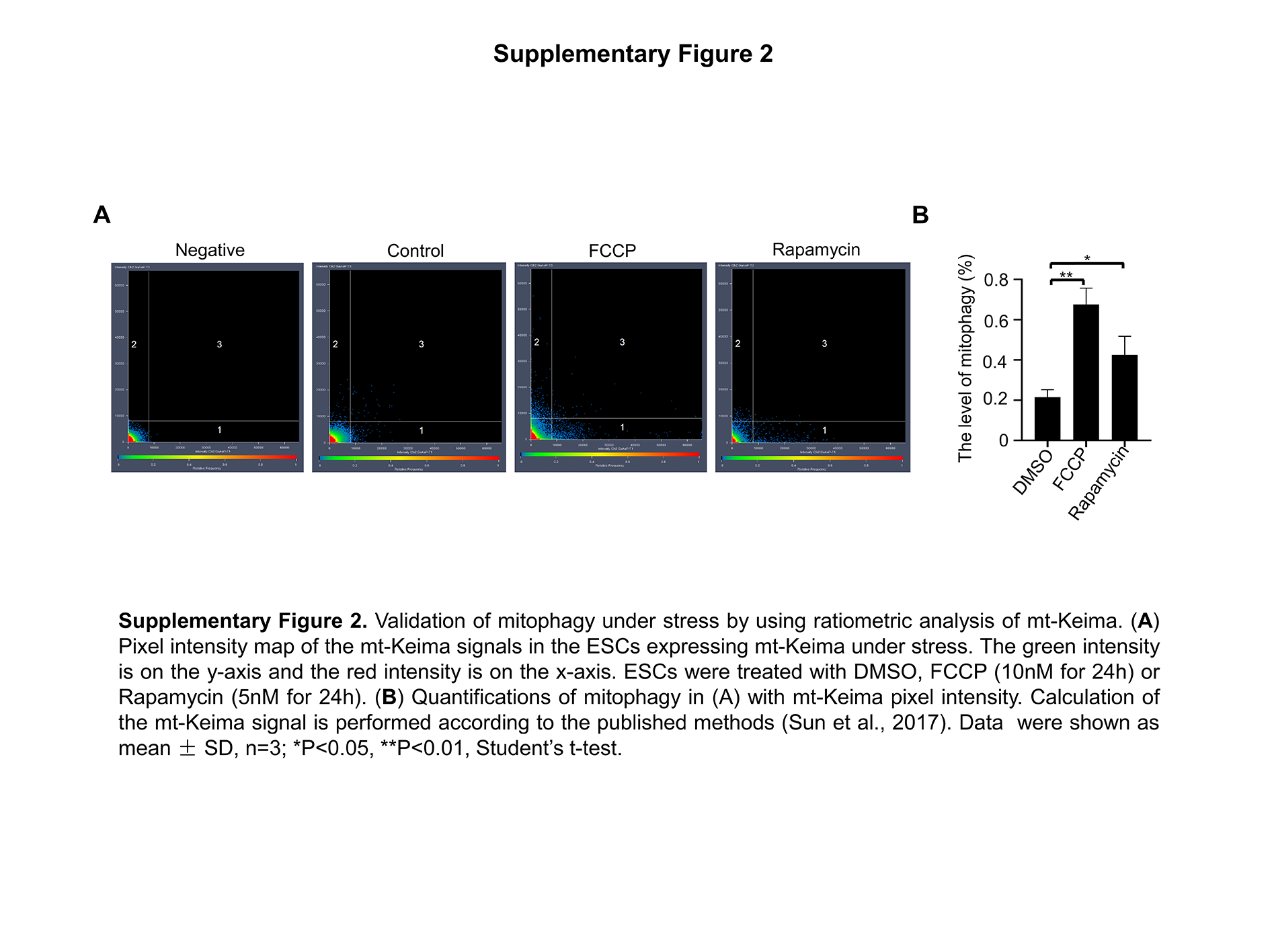

Supplement: Supplementary file 2 [file Image2.TIF]

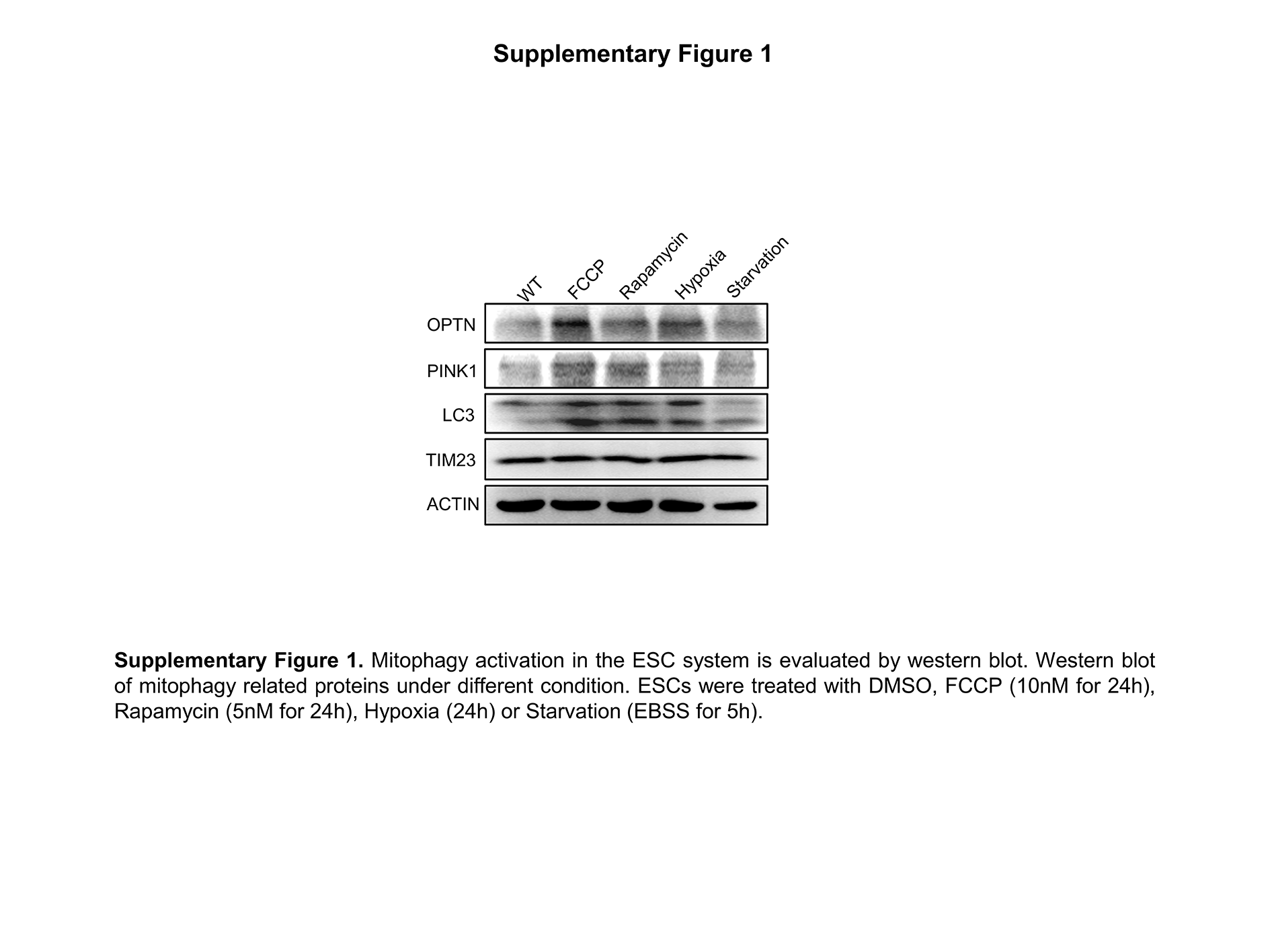

Supplement: Supplementary file 3 [file Image1.TIF]
